# Monotonicity properties for a ratio of finite many gamma functions

**DOI:** 10.1186/s13662-020-02655-4

**Published:** 2020-05-01

**Authors:** Feng Qi, Dongkyu Lim

**Affiliations:** 1 Institute of Mathematics, Henan Polytechnic University, Jiaozuo, China; 2 College of Mathematics, Inner Mongolia University for Nationalities, Tongliao, China; 3 School of Mathematical Sciences, Tianjin Polytechnic University, Tianjin, China; 4 Department of Mathematics Education, Andong National University, Andong, Republic of Korea

## Abstract

In the paper, the authors consider a ratio of finite many gamma functions and find its monotonicity properties such as complete monotonicity, the Bernstein function property, and logarithmically complete monotonicity.

## Preliminaries

Let $f(x)$ be an infinite differentiable function on an infinite interval $(0,\infty )$.

1. If $(-1)^{k}f^{(k)}(x)\ge 0$ for all $k\ge 0$ and $x\in (0,\infty )$, then we call $f(x)$ a completely monotonic function on $(0,\infty )$. See the review papers [22, 31, 36] and [35, Chapter IV].
2. If $(-1)^{k}[\ln f(x)]^{(k)}\ge 0$ for all $k\ge 1$ and $x\in (0,\infty )$, or say, if the logarithmic derivative $[\ln f(x)]'=\frac{f'(x)}{f(x)}$ is a completely monotonic function on $(0,\infty )$, then we call $f(x)$ a logarithmically completely monotonic function on $(0,\infty )$. See the papers [3, 4, 7, 24] and [33, Chap. 5].
3. If $f'(x)$ is a completely monotonic function on $(0,\infty )$, then we call $f(x)$ a Bernstein function on $(0,\infty )$. See the paper [28] and the monograph [33].

The classical gamma function $\varGamma (z)$ can be defined by

$$ \varGamma (z)= \int _{0}^{\infty }t^{z-1}e^{-t} \, \mathrm{d} t, \quad \Re (z)>0 $$

or by

$$ \varGamma (z)=\lim_{n\to \infty }\frac{n!n^{z}}{\prod_{k=0}^{n}(z+k)}, \quad z\in \mathbb{C} \setminus \{0,-1,-2,\ldots \}. $$

See [1, Chap. 6], [15, Chap. 5], the paper [18], and [34, Chap. 3]. In the literature, the logarithmic derivative

$$ \psi (z)=\bigl[\ln \varGamma (x)\bigr]'=\frac{\varGamma '(z)}{\varGamma (z)} $$

and its first derivative $\psi '(z)$ are respectively called the digamma and trigamma functions. See the papers [5, 6, 10, 25, 26] and closely related references therein.

## Motivations

This paper is motivated by a series of papers [2, 11, 12, 16, 19, 21, 27, 29, 32]. For detailed review and survey, please read the papers [19, 27, 29, 32] and closely related references therein.

In the paper [2], motivated by [11, 12], the function

2.1 $$ x\in (0,\infty )\mapsto \frac{\varGamma (nx+1)}{\varGamma (kx+1)\varGamma ((m-k)x+1)} p^{kx}(1-p)^{(m-k)x} $$

was considered, where $p\in (0,1)$ and *k*, *m* are nonnegative integers with $0\le k\le m$.

In [16, Theorem 2.1] and [32], the function

2.2 $$ x\in (0,\infty )\mapsto \frac{\varGamma (1+x\sum_{i=1}^{m}\lambda _{i} )}{\prod_{i=1}^{m}\varGamma (1+x\lambda _{i})} \prod _{i=1}^{m}p_{i}^{x\lambda _{i}} $$

was independently studied, where $m\ge 2$, $\lambda _{i}>0$ for $1\le i\le m$, $p_{i}\in (0,1)$ for $1\le i\le m$, and $\sum_{i=1}^{m}p_{i}=1$. The *q*-analogue

2.3 $$ x\in (0,\infty )\mapsto \frac{\varGamma _{q} (1+x\sum_{i=1}^{m}\lambda _{i} )}{\prod_{i=1}^{m}\varGamma _{q}(1+x\lambda _{i})} \prod _{i=1}^{m}p_{i}^{x\lambda _{i}} $$

of the function in (2.2) was investigated in [19], where $q\in (0,1)$, $m\ge 2$, $\lambda _{i}>0$ for $1\le i\le m$, $p_{i}\in (0,1)$ for $1\le i\le m$ with $\sum_{i=1}^{m}p_{i}=1$, and $\varGamma _{q}$ is the *q*-analogue of the gamma function *Γ*.

The functions

2.4 $$ x\in (0,\infty )\mapsto \frac{\prod_{i=1}^{m}\varGamma (\nu _{i}x+1)\prod_{j=1}^{n}\varGamma (\tau _{j}x+1 )}{\prod_{i=1}^{m}\prod_{j=1}^{n}\varGamma (\lambda _{ij}x+1 )} $$

and

2.5 $$ x\in (0,\infty )\mapsto \frac{\prod_{i=1}^{m}\varGamma (1+\nu _{i}x)\prod_{j=1}^{n}\varGamma (1+\tau _{j}x )}{ [\prod_{i=1}^{m}\prod_{j=1}^{n}\varGamma (1+\lambda _{ij}x ) ]^{\rho }} $$

were respectively considered in [17, Theorem 2.1] and [29, Theorem 4.1], where $\rho \in \mathbb{R}$ and $\lambda _{ij}>0$, $\nu _{i}=\sum_{j=1}^{n}\lambda _{ij}$, $\tau _{j}=\sum_{i=1}^{m}\lambda _{ij}$ for $1\le i\le m$ and $1\le j\le n$.

In [27], the function

2.6 $$ x\in (0,\infty )\mapsto \frac{\prod_{i=1}^{m}[\varGamma (1+\nu _{i}x)]^{\nu _{i}^{\theta }} \prod_{j=1}^{n} [\varGamma (1+\tau _{j}x ) ]^{\tau _{j}^{\theta }}}{\prod_{i=1}^{m}\prod_{j=1}^{n} [\varGamma (1+\lambda _{ij}x ) ]^{\rho \lambda _{ij}^{\theta }}} $$

was discussed, where $\rho ,\theta \in \mathbb{R}$ and $\lambda _{ij}>0$, $\nu _{i}=\sum_{j=1}^{n}\lambda _{ij}$, $\tau _{j}=\sum_{i=1}^{m}\lambda _{ij}$ for $1\le i\le m$ and $1\le j\le n$.

In this paper, stimulated by the above six functions (2.1), (2.2), (2.3), (2.4), (2.5), and (2.6), we consider a new function

2.7 $$ \mathcal{Q}(x)=\mathcal{Q}_{m,a,p,\rho ,\varrho ,\theta }(x) = \frac{ [\varGamma (1+x\sum_{i=1}^{m}a_{i} ) ]^{(\sum _{i=1}^{m}a_{i})^{\theta }}}{\prod_{i=1}^{m}[\varGamma (1+xa_{i})]^{\rho a_{i}^{\theta }}} \Biggl( \prod_{i=1}^{m}p_{i}^{a_{i}} \Biggr)^{\varrho x} $$

on $(0,\infty )$, where $m\ge 2$, $\rho ,\varrho ,\theta \in \mathbb{R}$, $a=(a_{1},a_{2},\ldots ,a_{m})$ with $a_{i}>0$ for $1\le i\le m$, and $p=(p_{1},p_{2},\ldots ,p_{m})$ with $p_{i}\in (0,1)$ for $1\le i\le m$ and $\sum_{i=1}^{m}p_{i}=1$.

## Monotonicity properties

In this section, we now start out to find and prove some monotonicity properties for the function $\mathcal{Q}(x)=\mathcal{Q}_{m,a,p,\rho ,\varrho ,\theta }(x)$ defined in (2.7). Our main results in this section can be stated in the following theorem.

### Theorem 3.1

*Let*$m\ge 2$, $a=(a_{1},a_{2},\ldots ,a_{m})$*with*$a_{i}>0$*for*$1\le i\le m$, *and*$p=(p_{1},p_{2},\ldots , p_{m})$*with*$\sum_{i=1}^{m}p_{i}=1$*and*$p_{i}\in (0,1)$*for*$1\le i\le m$. *Then*

1. *when*$\rho \le 1$*and*$\theta \ge 0$, *the second logarithmic derivative*
  $$ \bigl[\ln \mathcal{Q}(x)\bigr]''= \Biggl(\sum _{i=1}^{m}a_{i} \Biggr)^{\theta +2} \psi ' \Biggl(1+x\sum_{i=1}^{m}a_{i} \Biggr) -\rho \sum_{i=1}^{m}a_{i}^{ \theta +2} \psi '(1+a_{i}x) $$
  *is completely monotonic on*$(0,\infty )$;
2. *when*$\rho =1$, $\varrho =0$, *and*$\theta =0$, *the function*
  $$ \mathcal{Q}_{m,a,p,1,0,0}(x)= \frac{\varGamma (1+x\sum_{i=1}^{m}a_{i} )}{\prod_{i=1}^{m}\varGamma (1+xa_{i})} $$
  *is increasing on*$(0,\infty )$*and its logarithmic derivative*
  $$ \bigl[\ln \mathcal{Q}_{m,a,p,1,0,0}(x)\bigr]'= \Biggl(\sum _{i=1}^{m}a_{i} \Biggr)\psi \Biggl(1+x\sum_{i=1}^{m}a_{i} \Biggr) -\sum_{i=1}^{m}a_{i} \psi (1+a_{i}x) $$
  *is a Bernstein function on*$(0,\infty )$;
3. *when*$\rho =1$, $\varrho \ge 1$, *and*$\theta =0$, *the function*$\mathcal{Q}_{m,a,p,1,\varrho ,0}(x)$*is logarithmically completely monotonic on*$(0,\infty )$;
4. *when*$(\rho ,\varrho ,\theta )\in S$*and*
  $$ S=\{\rho \le 1,\varrho \ge 0,\theta \ge 0\}\setminus \{\rho =1, \varrho =0, \theta =0\}\setminus \{\rho =1,\varrho \ge 1,\theta =0\}, $$
  *the function*$\mathcal{Q}_{m,a,p,\rho ,\varrho ,\theta }(x)$*has a unique minimum on*$(0,\infty )$.

### Proof

Direct calculation gives

$$\begin{aligned}& \ln \mathcal{Q}(x)= \Biggl(\sum_{i=1}^{m}a_{i} \Biggr)^{\theta }\ln \varGamma \Biggl(1+x\sum_{i=1}^{m}a_{i} \Biggr) -\rho \sum_{i=1}^{m}a_{i}^{\theta } \ln \varGamma (1+a_{i}x) +\varrho x\sum_{i=1}^{m}a_{i} \ln p_{i}, \\& \bigl[\ln \mathcal{Q}(x)\bigr]'= \Biggl(\sum _{i=1}^{m}a_{i} \Biggr)^{\theta +1} \psi \Biggl(1+x\sum_{i=1}^{m}a_{i} \Biggr) -\rho \sum_{i=1}^{m}a_{i}^{ \theta +1} \psi (1+a_{i}x) +\varrho \sum_{i=1}^{m}a_{i} \ln p_{i}, \end{aligned}$$

and

$$ \bigl[\ln \mathcal{Q}(x)\bigr]''= \Biggl(\sum _{i=1}^{m}a_{i} \Biggr)^{\theta +2} \psi ' \Biggl(1+x\sum_{i=1}^{m}a_{i} \Biggr) -\rho \sum_{i=1}^{m}a_{i}^{ \theta +2} \psi '(1+a_{i}x). $$

As in [27, 29, 32], from

$$ \psi '(z)= \int _{0}^{\infty }\frac{t}{1-e^{-t}}e^{-zt}\, \mathrm{d} t, \quad \Re (z)>0 $$

in [1, p. 260, 6.4.1], it follows that

$$ \psi '(1+\tau z)= \int _{0}^{\infty }\frac{t}{1-e^{-t}}e^{-(1+\tau z)t} \, \mathrm{d} t =\frac{1}{\tau } \int _{0}^{\infty }h \biggl(\frac{v}{\tau } \biggr)e^{-vz} \,\mathrm{d} v, $$

where $\tau >0$ and $h(t)=\frac{t}{e^{t}-1}$ is the generating function of the classical Bernoulli numbers, see [20, 23] and [34, Chap. 1]. Accordingly, we have

3.1 $$ \bigl[\ln \mathcal{Q}(x)\bigr]''= \int _{0}^{\infty } \Biggl[ \Biggl(\sum _{i=1}^{m}a_{i} \Biggr)^{\theta +1}h \biggl(\frac{v}{\sum_{i=1}^{m}a_{i}} \biggr) - \rho \sum_{i=1}^{m}a_{i}^{\theta +1}h \biggl(\frac{v}{a_{i}} \biggr) \Biggr]e^{-vx}\,\mathrm{d} v. $$

In [27, Theorem 4.1], it was discovered that

3.2 $$ \sum_{i=1}^{m} \frac{\nu _{i}^{\alpha }}{e^{x/\nu _{i}}-1}+ \sum_{j=1}^{n} \frac{\tau _{j}^{\alpha }}{e^{x/\tau _{j}}-1} \ge 2\sum_{i=1}^{m}\sum _{j=1}^{n} \frac{\lambda _{ij}^{\alpha }}{e^{x/\lambda _{ij}}-1}, $$

where $\alpha \ge 0$, $x>0$, $\lambda _{ij}>0$ for $1\le i\le m$ and $1\le j\le n$, $\nu _{i}=\sum_{j=1}^{n}\lambda _{ij}$, and $\tau _{j}=\sum_{i=1}^{m}\lambda _{ij}$. As remarked in [27, Remark 4.1], setting $n=m$ and $\lambda _{1k}=\lambda _{k1}=\lambda _{k}>0$ for $1\le k\le m$ and letting $\lambda _{ij}\to 0^{+}$ for $2\le i,j\le m$ in inequality (3.2) result in

3.3 $$ \frac{ (\sum_{k=1}^{m}\lambda _{k} )^{\alpha }}{e^{x/\sum _{k=1}^{m}\lambda _{k}}-1} \ge \sum_{k=1}^{m} \frac{\lambda _{k}^{\alpha }}{e^{x/\lambda _{k}}-1} $$

for $x>0$, $\lambda _{k}>0$, and $\alpha \ge 0$. Inequality (3.3) can be equivalently formulated as

3.4 $$ \Biggl(\sum_{k=1}^{m} \lambda _{k} \Biggr)^{\alpha +1} h \biggl( \frac{x}{\sum_{k=1}^{m}\lambda _{k}} \biggr) \ge \sum_{k=1}^{m} \lambda _{k}^{\alpha +1} h \biggl(\frac{x}{\lambda _{k}} \biggr) $$

for $x>0$, $\lambda _{k}>0$, and $\alpha \ge 0$.

Combining inequality (3.4) with equation (3.1) yields that, when $\rho \le 1$ and $\theta \ge 0$, the second derivative $[\ln \mathcal{Q}(x)]''$ is completely monotonic on $(0,\infty )$.

The complete monotonicity of $[\ln \mathcal{Q}(x)]''$ implies that the first derivative $[\ln \mathcal{Q}(x)]'$ is strictly increasing on $(0,\infty )$. Therefore, by virtue of the limit

$$ \lim_{x\to \infty }\bigl[\ln x-\psi (x)\bigr]=0 $$

in [8, Theorem 1] and [9, Sect. 1.4], we have

$$\begin{aligned} \lim_{x\to 0^{+}}\bigl[\ln \mathcal{Q}(x)\bigr]'&=\lim _{x\to 0^{+}} \Biggl[ \Biggl(\sum_{i=1}^{m}a_{i} \Biggr)^{\theta +1}\psi \Biggl(1+x\sum_{i=1}^{m}a_{i} \Biggr) -\rho \sum_{i=1}^{m}a_{i}^{\theta +1} \psi (1+a_{i}x) \Biggr] \\ &\quad{} +\varrho \sum_{i=1}^{m}a_{i} \ln p_{i} \\ &=\psi (1) \Biggl[ \Biggl(\sum_{i=1}^{m}a_{i} \Biggr)^{\theta +1}-\rho \sum_{i=1}^{m}a_{i}^{\theta +1} \Biggr]+\varrho \sum_{i=1}^{m}a_{i} \ln p_{i} \\ &\textstyle\begin{cases} =0, & \theta =0,\rho =1,\varrho =0; \\ < 0, & \theta =0,\rho =1,\varrho >0; \\ < 0, & \theta =0,\rho < 1,\varrho \ge 0; \\ < 0, & \theta >0,\rho \le 1,\varrho \ge 0; \end{cases}\displaystyle \end{aligned}$$

where $\psi (1)=-0.577\ldots $ , and

$$\begin{aligned} \lim_{x\to \infty }\bigl[\ln \mathcal{Q}(x)\bigr]'&=\lim _{x\to \infty } \Biggl[ \Biggl(\sum_{i=1}^{m}a_{i} \Biggr)^{\theta +1}\psi \Biggl(1+x \sum_{i=1}^{m}a_{i} \Biggr) -\rho \sum_{i=1}^{m}a_{i}^{\theta +1} \psi (1+a_{i}x) \Biggr] \\ &\quad{} +\varrho \sum_{i=1}^{m}a_{i} \ln p_{i} \\ &=\lim_{x\to \infty } \Biggl\{ \Biggl(\sum _{i=1}^{m}a_{i} \Biggr)^{ \theta +1} \Biggl[\psi \Biggl(1+x\sum_{i=1}^{m}a_{i} \Biggr)-\ln \Biggl(1+x\sum_{i=1}^{m}a_{i} \Biggr) \Biggr] \\ &\quad{} -\rho \sum_{i=1}^{m}a_{i}^{\theta +1} \bigl[\psi (1+a_{i}x)-\ln (1+a_{i}x)\bigr] \Biggr\} +\varrho \sum_{i=1}^{m}a_{i}\ln p_{i} \\ &\quad{} +\lim_{x\to \infty } \Biggl[ \Biggl(\sum _{i=1}^{m}a_{i} \Biggr)^{ \theta +1}\ln \Biggl(1+x\sum_{i=1}^{m}a_{i} \Biggr) -\rho \sum_{i=1}^{m}a_{i}^{ \theta +1} \ln (1+a_{i}x) \Biggr] \\ &=\varrho \sum_{i=1}^{m}a_{i} \ln p_{i}+\lim_{x\to \infty }\ln \frac{ (1+x\sum_{i=1}^{m}a_{i} )^{(\sum _{i=1}^{m}a_{i})^{\theta +1}}}{\prod_{i=1}^{m}(1+a_{i}x)^{\rho a_{i}^{\theta +1}}} \\ &=\ln \lim_{x\to \infty } \frac{ (\frac{1}{x}+\sum_{i=1}^{m}a_{i} )^{(\sum _{i=1}^{m}a_{i})^{\theta +1}}}{\prod_{i=1}^{m} (\frac{1}{x}+a_{i} )^{\rho a_{i}^{\theta +1}}} \\ &\quad{} +\ln \lim_{x\to \infty }x^{(\sum _{i=1}^{m}a_{i})^{\theta +1}- \rho \sum _{i=1}^{m}a_{i}^{\theta +1}}+\varrho \sum _{i=1}^{m}a_{i} \ln p_{i} \\ &=\varrho \sum_{i=1}^{m}a_{i} \ln p_{i}+\ln \frac{ (\sum_{i=1}^{m}a_{i} )^{(\sum _{i=1}^{m}a_{i})^{\theta +1}}}{ (\prod_{i=1}^{m}a_{i}^{a_{i}^{\theta +1}} )^{\rho }} \\ &\quad{} + \textstyle\begin{cases} 0, &\rho = \frac{(\sum_{i=1}^{m}a_{i})^{\theta +1}}{\sum_{i=1}^{m}a_{i}^{\theta +1}}; \\ -\infty , &\rho > \frac{(\sum_{i=1}^{m}a_{i})^{\theta +1}}{\sum_{i=1}^{m}a_{i}^{\theta +1}}; \\ \infty , &\rho < \frac{(\sum_{i=1}^{m}a_{i})^{\theta +1}}{\sum_{i=1}^{m}a_{i}^{\theta +1}}. \end{cases}\displaystyle \end{aligned}$$

Let $\xi =(\xi _{1},\xi _{2},\ldots ,\xi _{m})$ such that $\sum_{i=1}^{m}\xi _{i}=1$ and $\xi _{i}\in (0,1)$ for $1\le i\le m$ and $m\ge 2$. Then the first derivative of the function $f(x)=\sum_{i=1}^{m}\xi _{i}^{x}$ is $f'(x)=\sum_{i=1}^{m}\xi _{i}^{x}\ln \xi _{i}<0$, which implies that the function $f(x)$ is strictly decreasing on $(-\infty ,\infty )$. Since $f(1)=1$, it follows that $f(x)\lesseqqgtr 1$ if and only if $x\gtreqqless 1$. This means that

$$ \sum_{i=1}^{m}\xi _{i}^{x} \lesseqqgtr 1, \quad x\gtreqqless 1. $$

Replacing $\xi _{i}=\frac{a_{i}}{\sum_{i=1}^{m}a_{i}}$ and $x=\theta +1$ in the above inequality yields

$$ \sum_{i=1}^{m} \biggl(\frac{a_{i}}{\sum_{i=1}^{m}a_{i}} \biggr)^{ \theta +1}\lesseqqgtr 1, \quad \theta \gtreqqless 0. $$

This can be further rewritten as

3.5 $$ \sum_{i=1}^{m}a_{i}^{\theta +1} \lesseqqgtr \Biggl(\sum_{i=1}^{m}a_{i} \Biggr)^{\theta +1}, \quad \theta \gtreqqless 0, a_{i}>0, m\ge 2. $$

Considering inequality (3.5) reveals that

1. when $\theta =0$, we have
  $$ \lim_{x\to \infty }\bigl[\ln \mathcal{Q}(x)\bigr]'=\varrho \sum_{i=1}^{m}a_{i} \ln p_{i}+ \textstyle\begin{cases} \ln \frac{ (\sum_{i=1}^{m}a_{i} )^{\sum _{i=1}^{m}a_{i}}}{\prod_{i=1}^{m}a_{i}^{a_{i}}}+0, &\rho =1; \\ \ln \frac{ (\sum_{i=1}^{m}a_{i} )^{\sum _{i=1}^{m}a_{i}}}{ (\prod_{i=1}^{m}a_{i}^{a_{i}} )^{\rho }}+ \infty , &\rho < 1. \end{cases} $$
2. when $\theta >0$ and $\rho \le 1$, we have
  $$ \lim_{x\to \infty }\bigl[\ln \mathcal{Q}(x)\bigr]'=\varrho \sum_{i=1}^{m}a_{i} \ln p_{i}+ \ln \frac{ (\sum_{i=1}^{m}a_{i} )^{(\sum _{i=1}^{m}a_{i})^{\theta +1}}}{ (\prod_{i=1}^{m}a_{i}^{a_{i}^{\theta +1}} )^{\rho }}+ \infty =\infty . $$

Hence, when $\theta =0$ and $\rho <1$ or when $\theta >0$ and $\rho \le 1$, we obtain

$$ \lim_{x\to \infty }\bigl[\ln \mathcal{Q}_{m,a,p,\rho ,\varrho ,\theta }(x) \bigr]'= \infty ; $$

when $\theta =0$ and $\rho =1$, we have

$$\begin{aligned} \lim_{x\to \infty }\bigl[\ln \mathcal{Q}(x)\bigr]' &= \varrho \sum_{i=1}^{m}a_{i} \ln p_{i}+ \ln \frac{ (\sum_{i=1}^{m}a_{i} )^{\sum _{i=1}^{m}a_{i}}}{\prod_{i=1}^{m}a_{i}^{a_{i}}} \\ &=(\varrho -1)\sum_{i=1}^{m}a_{i} \ln p_{i}+ \Biggl(\sum_{i=1}^{m}p_{i} \frac{a_{i}}{p_{i}} \Biggr) \ln \Biggl(\sum_{i=1}^{m}p_{i} \frac{a_{i}}{p_{i}} \Biggr) -\sum_{i=1}^{m}p_{i} \frac{a_{i}}{p_{i}} \ln \frac{a_{i}}{p_{i}}. \end{aligned}$$

Let *f* be a convex function on an interval $I\subseteq \mathbb{R}$, let $m\ge 2$ and $x_{i}\in I$ for $1\le i\le m$, and let $q_{i}>0$ for $1\le i\le m$. Then

3.6 $$ f \Biggl(\frac{1}{\sum_{i=1}^{m}q_{i}}\sum_{i=1}^{m}q_{i}x_{i} \Biggr)\le \frac{1}{\sum_{i=1}^{m}q_{i}}\sum_{i=1}^{m}q_{i}f(x_{i}). $$

This inequality is called Jensen’s discrete inequality for convex functions in the literature [13, Sect. 1.4] and [14, Chapter I]. Applying (3.6) to $f(x)=x\ln x$ which is convex on $(0,\infty )$, $x_{i}=\frac{a_{i}}{p_{i}}$, and $q_{i}=p_{i}$ leads to

$$ \Biggl(\sum_{i=1}^{m}p_{i} \frac{a_{i}}{p_{i}} \Biggr) \ln \Biggl( \sum_{i=1}^{m}p_{i} \frac{a_{i}}{p_{i}} \Biggr) \le \sum_{i=1}^{m}p_{i} \frac{a_{i}}{p_{i}}\ln \frac{a_{i}}{p_{i}}. $$

Accordingly,

$$ \lim_{x\to \infty }\bigl[\ln \mathcal{Q}(x)\bigr]'\le ( \varrho -1)\sum_{i=1}^{m}a_{i} \ln p_{i}\le 0, \quad \varrho \ge 1. $$

Consequently, when $\theta =0$, $\rho =1$, and $\varrho \ge 1$, the function $\mathcal{Q}_{m,a,p,\rho ,\varrho ,\theta }(x)$ is logarithmically completely monotonic on $(0,\infty )$.

The limit

$$ \lim_{x\to 0^{+}}\bigl[\ln \mathcal{Q}_{m,a,p,1,0,0}(x) \bigr]'=0 $$

obtained above implies that $[\ln \mathcal{Q}_{m,a,p,1,0,0}(x)]'\ge 0$, $\mathcal{Q}_{m,a,p,1,0,0}(x)$ is increasing, and then $[\ln \mathcal{Q}_{m,a,p,1,0,0}(x)]'$ is a Bernstein function on $(0,\infty )$.

When $(\rho ,\varrho ,\theta )\in S$, the limits

$$ \lim_{x\to 0^{+}}\bigl[\ln \mathcal{Q}_{m,a,p,\rho ,\varrho ,\theta }(x) \bigr]'< 0 $$

and

$$ \lim_{x\to \infty }\bigl[\ln \mathcal{Q}_{m,a,p,\rho ,\varrho ,\theta }(x) \bigr]'= \infty $$

derived above mean that the first derivative $[\ln \mathcal{Q}_{m,a,p,\rho ,\varrho ,\theta }(x)]'$ has a unique zero on $(0,\infty )$, that is, the functions $\ln \mathcal{Q}_{m,a,p,\rho ,\varrho ,\theta }(x)$ and $\mathcal{Q}_{m,a,p,\rho ,\varrho ,\theta }(x)$ have a unique minimum on $(0,\infty )$. The proof of Theorem 3.1 is complete. □

## An open problem

Let $m,n\ge 2$, $\rho ,\varrho ,\theta \in \mathbb{R}$, let $\lambda =(\lambda _{ij})_{m\times n}$ with $\lambda _{ij}>0$ for $1\le i\le m$ and $1\le j\le n$, let $\nu _{i}=\sum_{j=1}^{n}\lambda _{ij}$ and $\tau _{j}=\sum_{i=1}^{m}\lambda _{ij}$ for $1\le i\le m$ and $1\le j\le n$, and let $p=(p_{ij})_{m\times n}$ with $\sum_{i=1}^{m}\sum_{j=1}^{n}p_{ij}=1$ and $p_{ij}\in (0,1)$ for $1\le i\le m$ and $1\le j\le n$. Define

4.1 $$ Q_{m,n;\lambda ;p;\rho ;\varrho ;\theta }(x)= \frac{\prod_{i=1}^{m}[\varGamma (1+\nu _{i}x)]^{\nu _{i}^{\theta }} \prod_{j=1}^{n} [\varGamma (1+\tau _{j}x ) ]^{\tau _{j}^{\theta }}}{\prod_{i=1}^{m}\prod_{j=1}^{n} [\varGamma (1+\lambda _{ij}x ) ]^{\rho \lambda _{ij}^{\theta }}} \Biggl(\prod _{i=1}^{m}p_{ij}^{\lambda _{ij}} \Biggr)^{\varrho x} $$

on the infinite interval $(0,\infty )$.

Can one find monotonicity properties for the function $Q_{m,n;\lambda ;p;\rho ;\varrho ;\theta }(x)$ defined in equation (4.1)?

### Remark 4.1

This paper is a slightly revised version of the electronic preprint [30].
